# Body odor samples from infants and post-pubertal children differ in their volatile profiles

**DOI:** 10.1038/s42004-024-01131-4

**Published:** 2024-03-21

**Authors:** Diana Owsienko, Lisa Goppelt, Katharina Hierl, Laura Schäfer, Ilona Croy, Helene M. Loos

**Affiliations:** 1 Chair of Aroma and Smell Research, Friedrich-Alexander-Universität Erlangen-Nürnberg (FAU), Erlangen, Germany; 2 Department of Psychotherapy and Psychosomatics, Technical University of Dresden, Dresden, Germany; 3 Department of Clinical Psychology, Friedrich-Schiller-University of Jena, Jena, Germany; 4 Fraunhofer Institute for Process Engineering and Packaging IVV, Freising, Germany

**Keywords:** Bioanalytical chemistry, Chemical ecology, Mass spectrometry

## Abstract

Body odors change during development, and this change influences the interpersonal communication between parents and their children. The molecular basis for this chemical communication has not been elucidated yet. Here, we show by combining instrumental and sensory analyses that the qualitative odorant composition of body odor samples is similar in infants (0-3 years) and post-pubertal children (14-18 years). The post-pubertal samples are characterized by higher odor dilution factors for carboxylic acids and by the presence of 5α-androst-16-en-3-one and 5α-androst-16-en-3α-ol. In addition to the olfaction-guided approach, the compounds 6-methylhept-5-en-2-one (6MHO), geranyl acetone (GA) and squalene (SQ) were quantified. Both age groups have similar concentrations of 6MHO and GA, whereas post-pubertal children tend to have higher concentration of SQ. In conclusion, sexual maturation coincides with changes to body odor chemical composition. Whether those changes explain differences in parental olfactory perception needs to be determined in future studies with model odors.

## Abstract (web summary)

Body odor (BO) changes during human development, however, the molecular basis for this has not been fully elucidated. Here, the authors investigate the odorant composition of BO samples in infants and post-pubertal children, revealing qualitative and quantitative differences in the composition of the samples.

## Introduction

Chemosensory information conveyed by body odors (BOs) can play a role in social relationships, whether between friends^1,2^, partners^3,4^, or within families^5,6^. BOs contribute differently to interpersonal communication between parents and their offspring at different stages of development. Already shortly after birth, infants learn to recognize their mother´s individual odor^7–10^ and parents are able to identify their own infant’s BO, which is preferred over the smell of other infants^11–13^. BOs of infants are pleasant and rewarding to mothers^14,15^, and, as such, probably facilitate parental affection. In contrast, BOs of pubertal children are rated as less pleasant and parents are unable to identify their own child during this developmental stage^12,16,17^. Some studies even show a parental aversion towards the BO of their opposite sex pubertal children, which may serve to prevent inbreeding^5,12^. BO composition thus changes during the stages of development. Chemosensory cues indicating a child’s developmental status^18^ are still unknown but can be expected to be related to the onset of physiological changes during puberty, notably the activation of apocrine and apoeccrine sweat glands in the axillary, perineal, genital and anogenital regions^19,20^ and increased sebum secretion^20–22^. For these reasons, sweat and sebum can be hypothesized to differ qualitatively and quantitatively in line with a child’s developmental status.

So far, only a few researchers have studied the composition of children´s BO. In a pilot study, Uebi et al.^23^ tentatively identified 31 compounds in samples obtained from infants’ heads, using MonoTrap silica beads for sampling of volatiles, followed by thermodesorption-comprehensive gas chromatography-time of flight-mass spectrometry (TD-GCxGC-ToF-MS) analysis. Whereas the proportion of several aldehydes (e.g., hexanal, heptanal, octanal, nonanal) did not differ between the analyzed samples, the proportions of the corresponding carboxylic acids (e.g., hexanoic acid, heptanoic acid, octanoic acid, nonanoic acid) were higher in samples obtained from 2–3 day-old infants (*n* = 3) compared to those of 1 h-old infants (*n* = 2). The reason may be oxidation processes that occur at birth but not *in utero*. Further, Lam et al.^24^ investigated the microbial basis of malodor production in pre-pubertal (5-9 years) and post-pubertal (15-18 years) children. The microbiome of the underarm, neck and head was sampled before and after exercise. For this purpose, sport activities, e.g., treadmill, indoor cycling, aerobics, and basketball, were performed for a duration of 4–5 h, including intervals of physical exercise of 10–15 min followed by 10–15 min breaks. Odor intensity and odor quality of the different body sites were rated by a professional perfumer, before and after exercise. Post-pubertal underarm odor was rated as having a higher intensity than underarm odor of pre-pubertal children (corresponding to findings by Schäfer et al.^17^). Pre-pubertal BOs were mainly described as sour while post-pubertals’ BOs were mainly described as sour/sulfurous. Subsequent gas chromatography-mass spectrometry (GC-MS) and gas chromatography-olfactometry (GC-O) experiments with pooled and incubated sweat samples of both age groups were conducted to identify malodor-associated volatiles. In general, similar GC-MS and GC-O patterns were found for pre-pubertal and post-pubertal children, and the odorants acetic acid and 3-methylbutanoic acid were more abundant in the post-pubertal samples. These two odorants seemed to be the main contributors to the sour BO. *Staphylococcus* species were primarily involved in the production of these odorants from precursors in sweat, whereas *Corynebacterium* species had a lower impact on malodor in that study.

In sum, previous studies have shown developmental differences in children’s BOs. Principles of axillary malodor formation in adults are well-known^25–28^. However, no one has yet directly compared the chemical composition of BOs from infants and post-pubertal children. To study changes in BOs over this more extended period of development, we characterized the BO profiles of infants (0-3 years) and post-pubertal children (14-18 years) by performing direct contact sampling with cotton pads in the axillary region, solvent extraction and subsequent GC-O and GC-MS analysis. In the first part of the study, samples were analyzed using GC-O. Results revealed that the qualitative composition of odorants was similar in both age groups, mainly dominated by aldehydes and carboxylic acids. In the post-pubertal samples, higher odor dilution factors (OD) of carboxylic acids and 5α-androst-16-en-3-one as well as 5α-androst-16-en-3α-ol were determined. In the second part of the study, the target compounds 6-methylhept-5-en-2-one (6MHO), geranyl acetone (GA), and squalene (SQ) were selected based on a pilot study and quantified by GC-MS. Concentrations of 6MHO and GA were similar for both age groups, SQ tended to be higher in post-pubertal samples. In conclusion, developmental changes go along with an altered BO composition.

## Results

We used two independent approaches to characterize the composition of the BO samples. The first approach (‘Identification of odor-active compounds’) aimed to identify odor-active compounds in the BO samples by GC-O and estimate their potential contribution to BO by applying OEDA (for the significance and the limitations of this approach, see Grosch (2001)^29^). After tentative identification by GC-O, subsequent GC-MS analysis was conducted to complete identification by recording mass spectra, if possible (see Table 1). GC-O allows detection of trace compounds with low odor thresholds, which can be below LOD of instrumental detection systems, and is therefore essential in odor analysis. The second approach (‘Quantification of target compounds’) aimed to quantify target compounds by GC-MS independently of the first approach, which were selected based on outcomes of pilot study 1 (see Supplementary Note 1 online) indicating a potential difference between age groups. Please see the subsections ‘BO donors’ and ‘BO sampling procedure’ of the Material and Methods section for a study overview.

**Table 1:** Odor-active compounds detected in the distillates of pooled body odor samples (BO) and room samples (room) of infants and post-pubertal children

Identified compound	Odor attribute^f^	RI FFAP	RI DB5	OD Factor			
				Infants		Post-pubertals	
				BO	room	BO	room
Octanal^a, b, c, d^	Soapy, citrus-like	1280	1005	6	6	3	12
Oct-1-en-3-one^a, c^	Mushroom-like	1296	980	5	2	3	4
Nonanal^a, b, c^	Soapy, citrus-like	1383	1103	4	6	3	12
Unknown^c, d, e^	Flowery, soapy	1431	–	43	45	n.d.	43
Decanal^a, b, c, d^	Soapy	1489	1203	5	5	4	6
(*E*)-Non-2-enal^a, b, c, d, e^	Fatty, cardboard-like	1523	1160	33	17	6	7
**Linalool**^**a, b**^	**Flowery**	**1534**	**1104**	**n.d**.	**11**	**3**	**43**
Undecanal^a, b, c, d, e^	Soapy, citrus-like, coriander-like	1590	1308	5	1	n.d.	1
**3-Methylbutanoic acid**^**a**^	**Cheesy**	**1650**	**859**	**1**	**n.d**.	**6**	**1**
(2*E*,4*E*)-Nona-2,4-dienal^a, d^	Fatty, nutty	1690	1212	1	3	1	1
Dodecanal^a, b, c, d, e^	Soapy, coriander-like	1702	1409	11	48	11	32
(2*E*,4*E*)-Deca-2,4-dienal^a, c^	Fatty, deep-fried	1801	1317	89	48	33	109
α-Isomethylionone^a, b, c, d, e^	Violet-like	1829	1477	85	1	3	3
**Geranyl acetone**^**a, b**^	**Soapy**	**1859**	**1455**	**23**	**86**	**86**	**87**
Unknown^d, e^	Soapy, coriander-like	1873	–	8	15	1	1
**Polysantol**^**a**^	**Sandalwood-like**	**1905**	**1513**	**25**	**6**	**12**	**86**
**Unknown**	**Fatty, fruity, coconut-like**	**1920**	–	**3**	**11**	**1**	**n.d**.
**β-Ionone**^**a, b**^	**Flowery, violet-like**	**1930**	**1488**	**43**	**133**	**91**	**27**
**2-Methylheptanoic acid**^**a**^	**Fruity, dried plum-like**	**1949**	**1141**	**24**	**n.d**.	**32**	**n.d**.
Unknown^e^	Soapy, coriander-like	1963	–	5	5	13	27
(*E*)-4,5-Epoxy-(*E*)-2-decenal^a, c, d, e^	Metallic	2005	1376	171	277	128	277
**Octanoic acid**^**a, b**^	**Musty, coriander-like, fatty**	**2041**	**1175**	**1**	**n.d**.	**14**	**5**
*p*-Cresol^a, c^	Fecal, horse stable-like	2078	1086	3	9	6	27
Unknown^c, d, e^	Sandalwood-like, perfume-like	2094	–	45	53	347	74
Sandranol^a, b, c, d, e^	Sandalwood-like	2156	1575	203	65	149	64
Patchouli alcohol^a, c^	Earthy	2161	1664	32	128	385	128
Sotolon^a, d, e^	Savory, celery-like	2185	1102	1	1	3	3
**Unknown**	**Fecal, earthy**	**2188**	–	**6**	**2**	**6**	**1**
**4-Ethyloctanoic acid**^**a**^	**Goat-like**	**2195**	**1319**	**64**	**3**	**384**	**11**
**Unknown**	**Soap-like, perfume-like**	**2256**	–	**87**	**3**	**17**	**9**
γ-Undecalactone^a, c, d^	Peach-like, soapy	2245	1574	24	69	128	107
**Unknown**	**Fecal, musty**	**2260**	–	**3**	**n.d**.	**2**	**n.d**.
**Unknown**	**Soapy, coriander-like**	**2294**	–	**11**	**4**	**21**	**1**
γ -Dodecalactone^a, c, d, e^	Peach-like, flowery	2364	1679	43	23	87	44
**Dodecanoic acid**^**a, b**^	**Wax-like, soapy**	**2472**	**1569**	**1**	**n.d**.	**171**	**n.d**.
Unknown^c, d^	Vanilla-like	2475	–	43	21	1	86
**Unknown**	**Vanilla-like**	**2559**	–	**n.d**.	**11**	**44**	**3**
Vanillin^a, b, c, d, e^	Vanilla-like	2563	1400	256	112	89	192
**Myristoleic acid**^**a, b**^	**Earthy, green/grassy, green bell pepper-like**	**2753**	**1793**	**3**	**n.d**.	**11**	**n.d**.
Raspberry ketone^a, c, d, e^	Raspberry-like	2999	1560	261	112	429	195
**5α-Androst-16-en-3-one**^**a**^	**Sweaty, urinal, musk-like**	**3089**	**2320**	**n.d**.	**n.d**.	**1**	**n.d**.
**5α-Androst-16-en-3α-ol**^**a**^	**Sandalwood-like, musk-like**	**3094**	**2281**	**n.d**.	**n.d**.	**25**	**n.d**.

*RI* retention index, *OD* odor dilution.

Three pools per age group were investigated. Only compounds detected in at least two out of three sampling pools are displayed. This way, we aimed to minimize the potential impact of individual variations and potential exogenous contaminations from one sampling pool. Odor attributes, RI and OD factors (average value of three pools) are given. If the odorant was not detected, the value 0 was assigned for calculation of average OD factors. Compounds are marked in bold if they have not been detected in perfume-free shower gel, detergent, or blank unworn cotton pads.

^a^Compound was tentatively identified by GC-O by comparison of odor quality and retention indices on DB-FFAP and DB-5 of reference compound.

^b^Mass spectrum was compared with reference compound/in-house library.

^c^Detected in blank unworn cotton pads.

^d^Detected in perfume-free shower gel.

^e^Detected in perfume-free detergent.

^f^According to flavor language of the Chair of Aroma and Smell Research.

n.d OD factor was not determined (no odor quality perceivable).

### Identification of odor-active compounds

In total, 42 odor-active compounds were detected in at least two out of three BO sample pools per age group. Out of these, 16 odorants were tentatively identified based on their retention index (RI) and odor quality in comparison with reference compounds, and 15 odorants were identified by additional comparison with mass spectra. Lastly, 11 compounds remained unidentified. Table 1 lists the (tentatively) identified odor-active compounds with their respective average OD factors (of the three sampling pools per age group) in the BO samples and in the room samples. The table also shows which of these substances were also detected in unexposed cotton pads, perfume-free shower gel and detergent. Among the detected odor-active compounds, aldehydes represented the largest class of substances, including octanal (soapy, citrus-like), nonanal (soapy, citrus-like), decanal (soapy), (*E*)-non-2-enal (fatty, cardboard-like), undecanal (soapy, citrus-like, coriander-like), dodecanal (soapy, coriander-like), (2*E*,4*E*)-nona-2,4-dienal (fatty, nutty), (2*E*,4*E*)-deca-2,4-dienal (fatty, deep-fried), and (*E*)-4,5-epoxy-(*E*)-2-decenal (metallic). These aldehydes were on average between OD 1 and OD 277, and no definite trend between age groups was observed. The second largest group of odorants consisted of carboxylic acids: 2-methylheptanoic acid (fruity, dried plum-like), dodecanoic acid (wax-like, soapy), and myristoleic acid (earthy, green/grassy, green bell pepper-like), all of which were exclusively detected in the BO samples (OD factor range: OD 1 to OD 171). Further, 3-methylbutanoic acid (cheesy), octanoic acid (musty, coriander-like, fatty), and 4-ethyloctanoic acid (goat-like) had higher OD factors in the BO samples than in the room samples (OD 1 to OD 384). All of these carboxylic acids were detected with higher OD factors in the post-pubertal samples compared to the infant samples. In the group of ketones, oct-1-en-3-one (mushroom-like), raspberry ketone (raspberry-like), α-isomethylionone (flowery, soapy), β-ionone (flowery, violet-like), and the monoterpene ketone geranyl acetone (soapy) were present within a range of OD 2 to OD 429. Among these, α-isomethylionone was perceived with a higher average OD factor in the infant BO samples compared to the post-pubertal BO samples. Whereas for the alcohols polysantol (sandalwood-like), sandranol (sandalwood-like), and the monoterpene alcohol linalool (flowery), ranging between OD 3 and OD 203, no obvious difference between age groups emerged, an unknown odor (sandalwood-like, perfume-like) and the sesquiterpene alcohol patchouli alcohol (earthy) were detected with a higher average OD factor in the post-pubertal BO samples. The lactones γ-undecalactone (peach-like, soapy), γ-dodecalactone (peach-like, flowery), and sotolone (celery-like) as well as the phenol-derivate *p*-cresol (fecal, horse stable-like) and benzaldehyde-derivate vanillin (vanilla-like), perceived in a range of OD 1 to OD 256, revealed no further trends between age groups. Last, for the two age groups, samples differed with regard to the odor-active steroids 5α-androst-16-en-3-one (sweaty, urinal, musk-like) and 5α-androst-16-en-3α-ol (sandalwood-like, musk-like), since these were exclusively detected in the post-pubertal samples.

### Quantification of target compounds

**Fig. 1:**
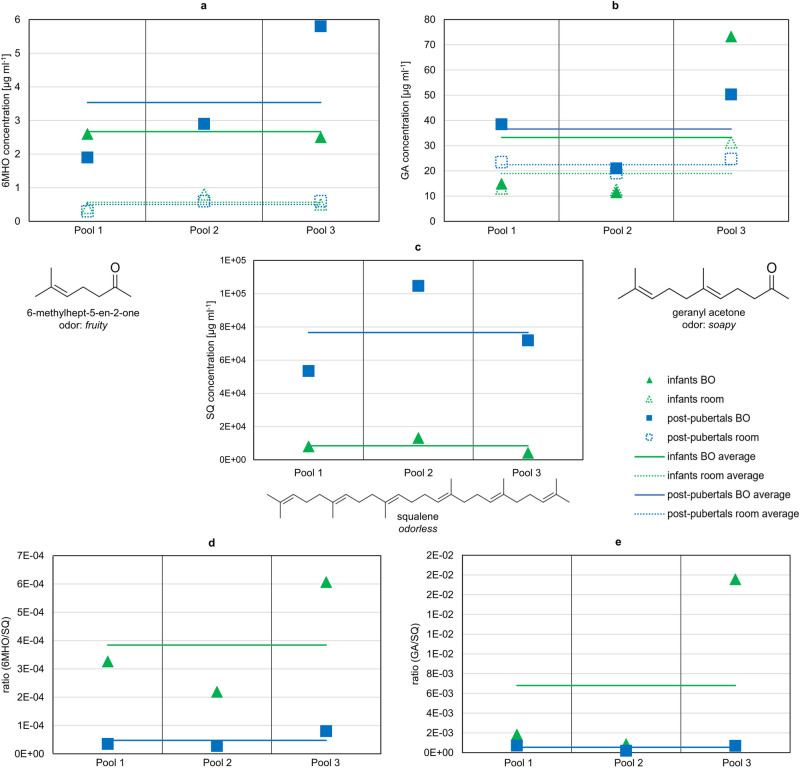
Quantification results of 6-methylhept-5-en-2-one (6MHO), geranyl acetone (GA), and squalene (SQ). Target compounds were quantified in the distillates of pooled body odor (BO) and room samples of infants (0–3 years) and post-pubertal children (14–18 years). In total, per age group three pools were analyzed including samples of six children each. Concentrations of (**a**) 6MHO, (**b**) GA, and (**c**) SQ in the BO and room samples of infants and post-pubertal children (see also Supplementary Note 2 Table S3 online); concentration ratios 6MHO/SQ and GA/SQ in BO samples are depicted in **d** and **e**. Note that SQ was out of calibration range in the room samples, due to considerably lower abundance than in the BO samples. For results of statistical comparison, see main text.

In infant BO samples, the average concentrations (±standard error), calculated from the three pools, of 6-methylhept-5-en-2-one (6MHO), geranyl acetone (GA), and squalene (SQ) were 2.7 ± 0.1 µg ml^−1^, 33.2 ± 20.1 µg ml^−1^, and 8.4 × 10^3^ ± 2.6 × 10^3 ^µg ml^−1^, respectively. In the corresponding room samples, 6MHO and GA occurred in concentrations of 0.6 ± 0.1 µg ml^−1^ and 18.9 ± 6.2 µg ml^−1^, respectively. Regarding the samples obtained from post-pubertal children, the average concentrations of 6MHO, GA, and SQ in the distillates were 3.5 ± 1.2 µg ml^−1^, 36.6 ± 8.5 µg ml^−1^, and 7.7 × 10^4^ ± 1.5 × 10^4 ^µg ml^−1^, respectively. In the room samples, 0.5 ± 0.1 µg ml^−1^ of 6MHO and 22.4 ± 1.7 µg ml^−1^ of GA were detected. For both age groups, SQ was detected > LOD in the room samples, however in considerably lower abundance than in the BO samples, thus the ratio of SQ to the internal standard was far beyond the calibration range. The results are graphically displayed in Fig. 1 and summarized in Supplementary Note 2 Table S3 online.

Comparing the absolute concentrations of 6MHO and GA between age groups, no significant difference emerged (exact Mann–Whitney *U*-test: *U* = 5.500, *p* = 0.70 and *U* = 6.000, *p* = 0.70, respectively). For SQ, the absolute concentration tended to be higher with strong effect in post-pubertal children compared to infant BO samples (*r* = 0.8; Mann–Whitney *U*-test: *U* = 9.000, *p* = 0.10).

Regarding comparison of BO and room samples (independent of age groups), concentration of 6MHO was significantly higher with strong effect in BO than in room samples (*r* = 0.8; Mann–Whitney U-test: *U* = 0.000, *p* = 0.002). For GA, comparison of BO and room samples revealed no significance (Mann–Whitney *U*-test: *U* = 13.000, *p* = 0.485).

Since 6MHO and GA are formed by oxidative degradation of SQ^30,31^, the ratios 6MHO/SQ and GA/SQ were calculated and averaged across the three sampling pools. For infants, average concentration ratios (±standard error) of 3.8 × 10^−4^ ± 1.2 × 10^−4^ and 6.8 × 10^−3^ ± 5.4 × 10^−3^ for 6MHO and GA, respectively, were determined. For post-pubertal children, the concentration ratio for 6MHO was 4.8 × 10^−5^ ± 1.6 × 10^−5^ and for GA 5.4 × 10^−4^ ± 1.7 × 10^−4^. The ratios of the three sampling pools are depicted in Fig. 1. Both ratios (6MHO/SQ, GA/SQ) tended to be higher in infant BO samples compared to post-pubertal BO samples with a strong effect (*r* = 0.8, 6MHO/SQ: Mann–Whitney *U*-test: *U* = 0.000, *p* = 0.10; GA/SQ: Mann–Whitney *U*-test: *U* = 0.000, *p* = 0.10).

In sum, the results show that 6MHO concentrations were higher in BO samples (independent of age group) than in room samples. Average concentrations of 6MHO and GA in BO samples were similar for both age groups, whereas SQ tended to be higher in post-pubertal BO samples. The 6MHO/SQ ratio and GA/SQ ratio both tended to be higher in infant BO samples than in post-pubertal BO samples.

## Discussion

In the present study BO samples of infants and post-pubertal children were comparatively analyzed by GC-O and GC-MS. Samples of both age groups were qualitatively similar in odorant composition, their odor-active compounds being mainly dominated by aldehydes and carboxylic acids. Nevertheless, two volatile steroids, 5α-androst-16-en-3-one and 5α-androst-16-en-3α-ol, were exclusive to the post-pubertal children. These odor-active steroids are well-known constituents of axillary odor and originate from apocrine sweat through microbial action^26,27,32–37^. They may contribute, together with other substances, to an altered quality of BO odor in post-pubertal children that became evident in our previous research^12,17^. In the present study, mass spectra could not be obtained for both steroids due to their low concentrations. Identification therefore remains tentative, based on retention indices and odor qualities on two different analytical columns. In previous work a recovery rate of 7% was determined for chemical analysis of 5α-androst-16-en-3-one when cotton pads were used as sampling material^38^, pointing to relatively high losses during sample workup. Nonetheless, despite the low recovery rate, both steroids could be clearly identified during GC-O analysis. The 2D-GC-MS system was used to enrich the eluting fraction that included both steroids; however, concentrations remained < LOD. This example shows the higher sensitivity of GC-O analysis for detecting trace compounds with low odor thresholds. To identify 5α-androst-16-en-3-one and 5α-androst-16-en-3α-ol through GC-MS, alternative analysis techniques may be tried in future studies^32,39^.

Besides the qualitative differences in odor-active compounds, carboxylic acids seemed to make a greater contribution to the overall BO of post-pubertal children compared to infants, as indicated by higher OD factors. Our results support the finding of Lam et al.^24^ that 3-methylbutanoic acid is more abundant in post-pubertal samples. However, it was not among the volatiles with the highest OD factors in the present study. Similarly, acetic acid was not detected in the present study, though previously described as the main influence on children´s BO by Lam et al.^24^ Among further carboxylic acids identified by Lam et al.^24^, only octanoic acid was additionally detected in the present study as having higher OD factors in the post-pubertal samples. Zeng and colleagues (1991)^33^ also report carboxylic acids as part of axillary odor. In line with the results obtained here, 2-methylheptanoic acid, octanoic acid and 4-ethyloctanoic acid were detected in their studies (in samples from men: Zeng et al. (1991)^33^, in samples from women: Zeng et al. (1996)^35^). In both studies (1991 and 1996) axillary secretions were sampled with cotton pads, however in the 1996 study of Zeng et al.^35^, apocrine secretion of female donors was sampled, additionally. In that study, only octanoic acid was identified, but not 4-ethyloctanoic acid and 2-methylheptanoic acid, perhaps because sebaceous glands may also be involved in secretion of the carboxylic acid precursors. Natsch et al.^40^ reported that 4-ethyloctanoic acid is secreted as a glutamine conjugate by axillary glands. However, the type of gland was not further specified. For future studies, it would be of interest to separately investigate the composition of children´s sebum and secretions of different sweat glands. In addition, it would be of interest to study BOs from other body parts. Here, we focused on axillary odor, but also other regions of the human body emit odor-active compounds and could thus contribute to social communication.

Approximately 15–30% of human sebum consists of free fatty acids^41,42^ and especially reaction of unsaturated fatty acids with ozone in ambient air leads to the formation of aldehydes as prominent volatiles^30,43,44^. This class of substances was also predominantly detected in our samples. Further oxidation may lead to the formation of their respective carboxylic acids as final products^44^. Considering these oxidative processes, it can be assumed that the more sebum is secreted, the higher is the abundance not only of long- but also of short- and medium-chained carboxylic acids. Further, not only the amount of initially secreted fatty acids, but also the exposure duration of these skin lipids to ambient air can be an influencing factor leading to a higher amount of carboxylic acids. The latter assumption of aging skin lipids can be strengthened by the results obtained by Uebi et al.^23^, who observed elevated amounts of carboxylic acids in the samples of 2–3 day old infants compared to 1 h-old infants, whereas respective aldehydes remained consistent. This observation and assumptions are also in line with our results, as a trend of increasing OD factors for carboxylic acids appeared in the post-pubertal samples compared to the infants, though not for corresponding aldehydes. For future studies, these GC-O results should be confirmed by quantitative analysis.

Exogenous compounds used as perfuming agents in cosmetic products or detergents were detected in the BO samples, e.g., linalool, α-isomethylionone, polysantol, β-ionone, sandranol, patchouli alcohol (European Commission database for information on cosmetic substances and ingredients (CosIng)^45^). Exogenous compounds in the human volatilome can hardly be avoided even with the hygienic and dietary protocols employed in the present study. Persistence of such perfuming odorants on human skin is demonstrated here. In addition, analysis of the perfume-free shower gel and detergent, which were provided to the participants, revealed the presence of odor-active compounds in these products. For those odorants, it is difficult to evaluate whether their origin was only in the cosmetic products or whether they also naturally occur on human skin. Another aspect to be considered is that nearly all of the detected compounds were perceived in the BO samples and also in the room samples. Of the (tentatively) identified compounds, only 2-methylheptanoic acid, myristoleic acid, 5α-androst-16-en-3-one, and 5α-androst-16-en-3α-ol were detected in the BO samples, exclusively. This finding indicates that most of the detected compounds are highly volatile, influence indoor air, and easily adhere to textiles (like cotton) when humans are residing in a room (see Weschler (2016)^44^ for a review). For some odorants, higher OD factors were determined in the room sample compared to the BO sample, confirming the omnipresence of some BO components in indoor air^46^. Besides being emitted by persons present in the rooms, such compounds can also originate from sebum residues such as SQ and skin lipids that remain on indoor surfaces and can be oxidized to volatile compounds, such as 6MHO, GA and aldehydes^44,46^. However, further exogenous origin is also conceivable, e.g., from furniture itself or other objects present in the room, or from the sampling material itself. In future studies, quantification should be carried out in room samples but also in unworn cotton pads not exposed to room air to further evaluate baseline levels of these compounds in the sampling material. Analysis of unworn cotton pads also led to detection of odor-active compounds, although pre-treated with solvent. Direct contact sampling with cotton textiles is often used in BO studies, especially for sensory evaluations^3,12,17,47,48^. As this research was closely linked to our previous work^12,17^, we decided to use the same BO sampling method in the present study. In future studies sampling of volatiles from indoor air should be controlled for when this sample material is used. For instance, for sensory evaluations, a blank or control textile could be presented to the rater to gain a first impression of the odor intensity of the sampling medium itself. In addition, future analytical studies should consider complementary or alternative methods, e.g., comparing the results of direct contact textile sampling with headspace sampling of skin volatile emissions. In addition, odor reconstitution models representing the odor of infants and post-pubertal children should be prepared and sensorially compared with real BO samples by a trained panel.

Unsaturated or hydroxylated branched fatty acids and sulfanylalkanols commonly described as typical constituents of axillary odors were not detected in our study, though their occurrence could be expected for the samples of post-pubertal children, as precursors for these compounds are secreted by apocrine sweat glands^26,28,34,35,49^. A possible reason for this observation is that sampling was conducted over night while sleeping and therefore no sufficient amount of these precursors might have been secreted. However, Lam et al.^24^ reported that 3-methyl-2-hexenoic acid and 3-hydroxy-3-methylhexanoic acid were not detected, as well, or only in minimal amounts, although sweat sampling was performed after exercise. A further possible explanation is that the here used sampling and extraction method might not enable detection of these compounds. For instance, it is known that cotton as sampling material is not suited for detection of trace amounts of certain compounds, e.g., sulfury components^38,50^. Determining recovery rates for these compounds could help clarify this issue in future studies.

In the second independent approach of our study, the target compounds 6MHO, GA and SQ were quantified via GC-MS based on pilot study 1, see Supplementary Note 1 online. SQ tended to occur in a higher amount (factor 9 higher) in the distillates of BO samples obtained from the post-pubertal compared to the younger age group of infants. Because SQ is one of the main components of human sebum (up to 12%)^42^ our finding may be due to androgenic stimulation of sebaceous glands during puberty^20^. Additionally, the percentage of SQ in sebum is higher in adults than in children (9.3–10.2% vs. 6.3%)^51^. In contact with ozone (present in ambient air) SQ is oxidized, leading to the formation of 6MHO and GA, inter alia^30,31,52^. Additionally, 6MHO and GA might originate from enzymatic activity on the skin. The relative concentrations of 6MHO and GA in relation to SQ, considered to be their precursor, tended to be higher in the infant BO samples than in the post-pubertal BO samples (factor 8 and factor 13 for 6MHO and GA, respectively). This finding may be explained by four different hypotheses. First, the amount of sweat on the skin may be an influencing factor for ozone-mediated reactions. Post-pubertal skin may be covered to a greater extent with aqueous sweat since apocrine and apoeccrine glands are additionally contributing to the overall amount of sweat and further sweating rate increases with maturation^53,54^. A higher humidity above the skin surface may lead to lower relative concentrations of SQ ozonolysis products (6MHO and GA). Relative humidity can affect squalene ozonolysis in several ways due to competing reactions of ozone with water vapor and shift of volatile reaction products^31^. Due to the complexity of squalene ozonolysis, and because we are not aware of any study comparing the amount of sweat in the axilla of infants vs the axilla of post-pubertals, further research is needed for clarification. Second, different degrees of skin bacterial colonization (or more generally different enzymatic activity on the skin) may lead to different degrees of SQ degradation products. Bacterial metabolization of SQ on human skin has not yet been investigated, to our knowledge. To prove bacterial action in SQ degradation, incubation of SQ with axillary skin bacteria should be conducted and oxidation intermediates of SQ investigated using LC-MS. Third, skin lipid composition may influence the oxidation rate of SQ. The preferred positions for ozone reactions are carbon-carbon double bonds. Because SQ has six double bonds and is distributed all over the human body, it is the most important compound for ozone reactions^44^. Unsaturated fatty acids are next in importance^44^ and their relative amounts change with aging^22^. Hence, SQ may be oxidized to a lesser extent if the proportion of unsaturated fatty acids in skin lipids is higher. This question may be resolved through experiments with different ratios of unsaturated fatty acids to SQ, with incubation at the same ozone concentrations. Additionally, further precursors of 6MHO and GA might be present and thereby influence their concentrations. Fourth, sebum production is higher in post-pubertal children than in infants, yet this higher level does not coincide with higher concentration of oxidation products, as detected here, e.g., because oxidation mainly occurs on the surface area or because of ad-/absorption equilibria of the textile sampling material.

During GC-O analysis, 6MHO (*fruity*) was not detected, whereas GA (*soapy*) was identified with intermediate OD factors. From these results, it appears that both compounds have a minor influence on BO. GC-O does not account for mixture effects, and the sampling technique may for some reason have discriminated against 6MHO and/or GA. Certain work-up losses occur for volatiles when extracted from cotton pads. For 6MHO the recovery rate was 37.9% when cotton pads were used^38^. To further evaluate the potential role of 6MHO and GA in BO perception, one should extract volatiles directly from the headspace above the skin and compare the concentrations to threshold concentrations in air. The thresholds in water (6MHO: 0.75 µg ml^−1^, GA: 0.43 µg ml^−1^) could be used in future studies to estimate concentrations in sweat that lead to odor detection of these compounds, but since the composition of sweat and sebum is more complex, additional matrix effects may occur.

Infant BO samples are rated to be more pleasant than BO samples from post-pubertal children (see also our pilot study 2, Supplementary Note 3 online), perhaps because the rather unpleasant smelling steroids are absent, but this awaits clarification by experiments with odor models. For future experiments it would also be of interest to test the physiological impact of 6MHO and GA when perceived by participants in sensory studies, for instance to observe whether they have a direct impact on the perception of children’s BO by parents.

Quantification data showed that 6MHO and GA were also detected in the room samples. When the standard errors of the BO samples and the room samples are compared, there is more variation within the BO samples, perhaps because of interpersonal variation in sebum secretion and seasonal variations. That variation may have two causes: i) higher ozone concentrations during warm and dry weather^55^ with higher SQ degradation; and ii) higher sebum and SQ secretion, respectively, as more sebum is secreted during warm temperatures^56^. Fruekilde et al.^52^ investigated ubiquitous occurrence of 6MHO and GA. According to their experiments the formation of 6MHO and GA can be traced back to the reaction of ozone with epicuticular waxes of leaves, which contain sesquiterpenoids (nerolidol; farnesol) and triterpenes (SQ) as major precursors. Additionally, they demonstrated formation of these compounds from skin lipids. In our sampling setup, cotton pads were carefully handled with gloves and not with bare fingers, e.g., during sewing or when they were cut out of the T-shirts/bodies. A human presence leads to higher concentrations of 6MHO, GA and to further increases in SQ degradation products in room air and also residues of SQ on indoor surfaces are further oxidized (reviewed by Weschler (2016)^44^, Coffaro & Weschler (2022)^31^). Therefore, 6MHO and GA may have also been present in the room samples.

## Materials and methods

### Chemicals

The following chemicals were used: sodium sulfate, dichloromethane (DCM) (VWR International GmbH, Darmstadt, Germany) - prior to use, DCM was freshly distilled; alkanes C_6_-C_34_, nonanal (purity: 95%) (Fluka, Steinheim, Germany); octanal (n.a.), oct-1-en-3-one (50%), (*E*)-non-2-enal (97%), undecanal (97%), dodecanoic acid (99,5%), dodecanal (n.a.), (2*E*,4*E*)-deca-2,4-dienal (85%), 3-methylbutanoic acid (99%), 4-ethyl octanoic acid (98%), 4-methylphenol (in the following: *p*-cresol; > 98%), octanoic acid (98%), 4-(4-hydroxyphenyl)butan-2-one (in the following: raspberry ketone; 99%), 5-heptyloxolan-2-one (in the following: γ-undecalactone; 98%), (2*E*,4*E*)-nona-2,4-dienal (85%), 4-hydroxy-2,3-dimethyl-2H-furan-5-one (in the following: sotolone; 97%) (Aldrich, Steinheim, Germany); 6-methylhept-5-en-2-one (>98%), (*E*)-3-methyl-4-(2,6,6-trimethylcyclohex-2-en-1-yl)but-3-en-2-one (in the following: α-isomethylionone; ≥95%), 6,10-dimethylundeca-5,9-dien-2-one (in the following: geranyl acetone; > 97%), 3,7-dimethylocta-1,6-dien-3-ol (in the following: linalool; 97%) (Sigma Aldrich, Steinheim, Germany); decanal (>98%), (3*R*,5*S*,8*R*,9*S*,10*S*,13*R*,14*S*)-10,13-dimethyl-2,3,4,5,6,7,8,9,11,12,14,15-dodecahydro-1H-cyclopenta[a]phenanthren-3-ol (in the following: 5α-androst-16-en-3α-ol; n.a.), (5*S*,8*R*,9*S*,10*S*,13*R*,14*S*)-10,13-dimethyl-1,2,4,5,6,7,8,9,11,12,14,15-dodecahydrocyclopenta[a]phenanthren-3-one (in the following: 5α-androst-16-en-3-one; n.a.), (*Z*)-tetradec-9-enoic acid (in the following: myristoleic acid; > 99%) (Sigma, Steinheim, Germany); (*E*)-2-ethyl-4-(2,2,3-trimethylcyclopent-3-en-1-yl)but-2-en-1-ol (in the following: sandranol; n.a.), (*E*)-3,3-dimethyl-5-(2,2,3-trimethylcyclopent-3-en-1-yl)pent-4-en-2-ol (in the following: polysantol; n.a.) (kindly provided by Symrise AG, Holzminden, Germany); (*E*)-3-[(2*S*,3*S*)-3-pentyloxiran-2-yl]prop-2-enal (in the following: (*E*)-4,5-epoxy-(*E*)-2-decenal; 97%) (AromaLab GmbH, Martinsried, Germany); 4-hydroxy-3-methoxybenzaldehyde (in the following: vanillin; 99%), 2-methylheptanoic acid (98%) (ABCR, Karlsruhe, Deutschland); 5-octyloxolan-2-one (in the following: γ-dodecalacton; 97%) (SAFC, Steinheim, Germany); (1*R*,3*R*,6*S*,7*S*,8*S*)-2,2,6,8-tetramethyltricyclo[5.3.1.03,8]undecan-3-ol (in the following: patchouli alcohol; > 98%) (Biozol, Eching, Germany); n-triacontane (>98%) (Alfa Aesar by Thermo Fisher Scientific, Kandel, Germany).

The following isotopically labeled standards were ordered: 6-methylhept-5-en-2-one d6, (2*E*)-3,7-dimethylocta-2,6-dien-1-ol d2 (in the following: geraniol d2; AromaLAB GmbH, Martinsried, Germany).

### BO donors

**Fig. 2:**
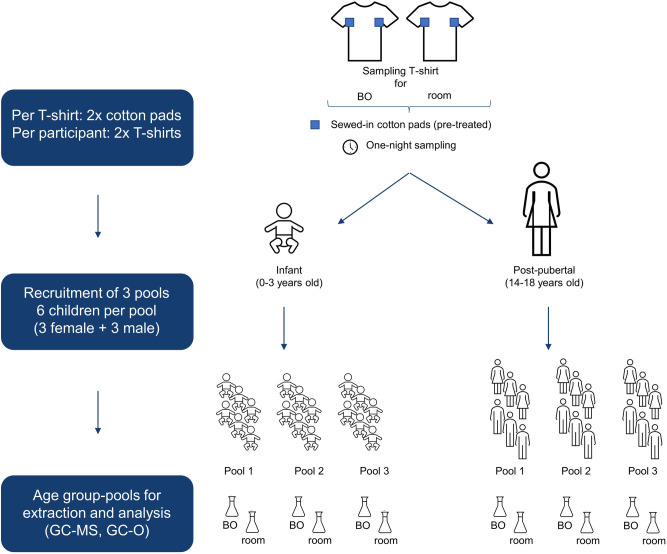
Study overview regarding preparation of sampling material, participants, and analysis. Cotton T-shirts with pre-treated and sewed-in cotton pads were used for body odor (BO) sampling of infants (0–3 years) and post-pubertal children (14–18 years). Additionally, a second T-shirt was placed in the room for air sampling during the BO sampling period (overnight). In total, three pools with six children each were recruited. For final analysis, cotton pads of BO and room samples were pooled, respectively, solvent extracted and analyzed by GC-MS and GC-O.

BOs were sampled from 18 healthy infants (9 girls, 9 boys; age (M ± SD): 1.3 ± 0.8 years) and 18 healthy post-pubertal children (9 girls, 9 boys; age (M ± SD): 15.5 ± 1.4 years). All children were of Caucasian ethnicity. As sampling took place during the Corona pandemic, all participants, or their respective parents, were queried for COVID-19 symptoms and carried out an olfactory test at home to minimize the potential infection risk mediated by the participants. Recruitment took place during July/August 2021, December 2021/January 2022, and April/May 2022 (resulting in three sampling pools), whereby each time 6 participants (3 girls, 3 boys) per age group were recruited (Fig. 2).

Written informed consent was provided by the children´s parents and – in case of post-pubertal children – additionally by the children themselves prior to the study. The study was conducted in accordance with the Declaration of Helsinki and approved by the Institutional Review Board of the Ethics Committee of the Dresden University of Technology (EK: 501122018).

### Cotton test fabric for sampling BO

Cotton test fabric (100%), following DIN 53919/ISO 2267, was purchased from wfk Testgewebe GmbH (Brüggen-Bracht, Germany). The fabric was delivered cut into 10 cm×10 cm patches. To achieve analytical cleanness, a pretreatment of the cotton test fabric with DCM (3 × 30 ml) was conducted according to Alves Soares et al.^38^.

### BO sampling procedure

For BO sampling pre-treated cotton patches were sewed in the left and right axillary region of 100% cotton T-Shirts and body suits (Fruit of the Loom Ltd, Bowling Green, KY, USA), which had been washed with perfume-free detergent (Denkmit Vollwaschmittel Ultra Sensitive, dm-drogerie markt GmbH & Co. KG, Karlsruhe, Germany). Participants received two of these prepared T-shirts/body suits. One T-shirt/body suit was worn by the BO donors for one night (BO sample). The other one was placed inside the room where the BO donors were sleeping, to sample volatile compounds which were present in the room air (room sample; Fig. 2). The post-pubertal children were instructed to follow a dietary and hygienic protocol during the 48 h before sampling – in case of the infants, the parents were instructed to ensure that the protocol was followed. Regarding diet, consumption of strongly spiced food, onions, garlic, leek and similar vegetables, asparagus, cabbage, and alcohol should be avoided. Regarding hygiene, perfumed hygienic products and deodorant should not be used. Clothes and bedlinen should be washed with perfume-free detergent (see above). Before the experimental night post-pubertal children were instructed to shower with perfume-free shower gel (Eubos Basic Care Liquid Washing Emulsion, Dr. Hobein (Nachf.) GmbH, Meckenheim, Germany). Analogously, parents were instructed to wash their infants. Perfume-free detergent and shower gel were handed out in an experimental kit. To record the sleeping situation, and the hygienic and dietary protocol, a questionnaire was filled out. After sampling was conducted, the two garments were placed in separate zip lock bags and returned to the experimenter within 8 h and frozen at -20 °C for a maximum of 62 days at the University Hospital Carl Gustav Carus, Dresden, Germany. For chemical analysis the samples were transported on dry ice to the Chair of Aroma and Smell Research, Friedrich-Alexander-Universität Erlangen-Nürnberg, Erlangen, Germany, where they were stored at -80 °C for a maximum of 15 days. To evaluate the impact of different transport conditions on the olfactory properties of BO samples, we performed a pilot test with BOs of a total of 16 participants (see Supplementary Note 3 online). Transport on dry ice was chosen due to its practicality.

### Isolation of volatiles from BO and room samples, and from perfume-free shower gel and detergent

The two sewed-in cotton patches were cut from each T-shirt/body suit. Samples from six participants per age group were pooled for extraction (12 patches in total). Overall, this resulted in three pools per age group. Room samples were pooled analogously (12 patches per pool). See Fig. 2 for sample pooling overview. For extraction of the volatiles 300 ml of DCM were added to the pooled cotton patches and for quantification purpose the (isotopically labeled) internal standards (see Supplementary Note 2 Table S1 online) were added. After stirring for 30 min at room temperature, the solvent was decanted and thereafter used for solvent-assisted flavor evaporation (SAFE)^57^, performed at 50 °C. The distillate was thawed at room temperature and dried with anhydrous sodium sulfate. In a last step, the distillate was concentrated to a volume of 100 µl by means of Vigreux distillation and microdistillation by Bemelmans^58^. Perfume-free detergent (0.11 g) and shower gel (0.14 g), which were provided to the participants for washing clothes and bedlinen and for showering, respectively, were extracted with 30 ml DCM and the extract was then distilled and concentrated analogously. The detergent was also used for washing T-shirt/body suits before handing them over to the participants.

### Gas chromatography-olfactometry

Measurements were conducted with a Trace GC Ultra (Thermo Fisher Scientific Inc., Waltham, USA) equipped with a DB-FFAP or DB-5 (30 m × 0.32 mm, film thickness of 0.25 µm, J&W Scientific, Agilent Technology, Santa Clara, CA, USA) as capillary column. The helium carrier gas flow rate was set at 2.5 ml min^−1^. After separation on the capillary column the gas flow was split 1:1 and directed to an olfactory detection port (ODP; 270 °C) and a flame ionization detector (FID; 250 °C). For recording of the chromatogram an electric writer Servogor 120 (BBC Goerz Metrawatt, Nürnberg, Germany) was used. Distillates were injected (2 µl) in cold-on-column mode. The initial oven temperature was set at 40 °C for 2 min, followed by a heating ramp of 10 °C min^−1^ to a final temperature of 240 °C with a hold time of 10 min and 300 °C with 5 min for DB-FFAP and DB-5, respectively.

Odor extract dilution analysis (OEDA) was performed by preparing dilutions 1:1 v/v with DCM. Odor dilution (OD) factors were determined at the dilution stage where the odor impression was last perceived. The diluted distillates were analyzed on a DB-FFAP column.

### Gas chromatography-mass spectrometry

GC-MS analysis was performed with a 7890 A GC equipped with a 5975 C MSD (Agilent, Santa Clara, CA, USA). For injection, a multipurpose autosampler MPS2 and CIS4 injection system (Gerstel GmbH & Co. KG, Mülheim an der Ruhr, Germany) were used. The injection was conducted in cold-on column mode with a volume of 1 µl. For GC separation a DB-FFAP and DB-5 column (30 m × 0.25 mm, film thickness of 0.25 µm, J&W Scientific, Agilent Technology, Santa Clara, CA, USA) were used. Helium carrier gas was set in constant flow at 1.0 ml min^−1^. The oven temperature started at 40 °C for 2 min, followed by a ramp of 8 °C min^−1^ to a final temperature of 240 °C for 10 min (FFAP) or 300 °C for 5 min (DB-5). Mass spectra were recorded with electron ionization (EI) at 70 eV in TIC (40-400 m/z) as well as SIM mode, see Supplementary Note 2 Table S1 online.

### Two-dimensional (heart cut) gas chromatography-mass spectrometry/olfactometry (2D-GC-MS/O)

The 2D-GC-MS/O analysis was performed with a system consisting of two 7890B GCs in combination with a 5977B MSD (Agilent Technologies, Santa Clara, California, USA). The sample was injected (1 µl) using a multipurpose autosampler MPS2 and a CIS4 injection system (Gerstel GmbH & Co.KG, Mülheim an der Ruhr, Germany) in cold-on column mode. The first GC was equipped with a multi-column switching system MCS2 and a cryo-trap system CTS1 connecting both GCs (Gerstel GmbH & Co.KG, Mülheim an der Ruhr, Germany). For the first oven a DB-FFAP column (30 m × 0.32 mm, film thickness of 0.25 µm, J&W Scientific, Agilent Technology, Santa Clara, CA, USA) and for the second oven a DB-5 column (30 m × 0.25 mm, film thickness of 0.25 µm, J&W Scientific, Agilent Technology, Santa Clara, CA, USA) was installed. Helium was used as a carrier gas (flow rate: 7.8 ml min^−1^). In the first oven the gas flow was split to a FID (250 °C) and ODP (280 °C). In addition, the effluent was led to the cryo trap during the cut interval and afterwards to the second oven, which was connected with the MSD as well as an ODP. The initial temperature program for the ovens was set at 40 °C for 2 min, followed by a heating ramp of 8 °C min^−1^ and a final temperature of 240 °C for 5 min (FFAP) or 300 °C for 5 min (DB-5). Mass spectra were recorded in EI mode at 70 eV in the range of 40–400 m/z.

### Identification of volatiles

In the first step, volatile and odor-active compounds in the distillates were tentatively identified by GC-O by comparison of retention indices (RIs) on both capillary columns (FFAP and DB-5) and odor impression of respective analytical standards, see Table 1 letter a. For calculation of RIs according to Kovats (1958)^59^ a homologous series of alkanes in the range of C6 to C34 was injected. If available, the mass spectrum was compared with the one of a standard, integrated into an in-house developed database established with AMDIS (Version 2.72, National Institute for Standards and Technology, Gaithersburg, USA) for identification of the compound, see Table 1 letter b.

### Determination of odor detection threshold of target compounds

Odor detection thresholds in distilled water were determined for 6MHO and GA by a trained panel (4 female, 2 male, M ( ± SD): 26.8 ± 2.7 years, age range: 23–30 years), applying triangle tests. For more information regarding the training of the panel, see Supplementary Note 3 online. For both compounds, six triangle tests were prepared, each consisting of two blank solutions (distilled water) and one target solution (odorant in water), which were filled into glass beakers (WECK Mini-Sturzglas, 140 mL, 60 mm diameter, J. Weck GmbH & Co. KG, Wehr-Öflingen, Germany). The concentration of the target solutions ranged from 0.25 to 60.86 µg ml^−1^ for 6MHO and from 0.02 to 20.00 µg ml^−1^ for GA. For each triangle test, the panelist sniffed the three beakers and indicated whether he/she detected a difference (forced choice decision). Individual thresholds were calculated by the geometric mean of the highest non-detected concentration and the lowest detected concentration after which all further tests were correctly detected. The average odor threshold for the panel was calculated by the geometric mean of all individual thresholds.

### Quantification of target compounds

The compounds 6-methylhept-5-en-2-one, geranyl acetone and squalene were quantified using 6-methylhept-5-en-2-one d6, geraniol d2 and triacontane as standards. These compounds were selected for quantification based on prior pilot tests (see Supplementary Note 1 online). Calibration solutions were prepared with target compound/internal standard ratios of approximately 5:1, 3:1. 1:1, 1:3, 1:5. If necessary, further ratios were prepared and measured. GC-MS measurements were conducted in selected ion monitoring (SIM) mode. The masses selected for each analyte and respective standard are listed in Supplementary Note 2 Table S1 online. For the calibration curves all coefficients of determination were above 0.99 (see Supplementary Note 2 Table S2 online). For limit of detection (LOD) and limit of quantification (LOQ) a signal-to-noise ratio of 3 and 10, respectively, was considered.

### Statistical analysis

Data were analyzed with IBM SPSS Statistics 25 (IBM Corp. Released 2017. IBM SPSS Statistics for Windows, Version 25.0. Armonk, NY: IBM Corp.). Mann-Whitney-U tests were conducted to compare results obtained for different age groups. The exact significance [2*(one-sided significance)] was reported.

### Reporting summary

Further information on research design is available in the Nature Portfolio Reporting Summary linked to this article.

### Supplementary information

Supplementary Information

Reporting Summary

## Data Availability

The datasets generated during and/or analyzed during the current study are available from the corresponding author on reasonable request.
