# Supplementary material for: Body odor samples from infants and post-pubertal children differ in their volatile profiles
Source: Commun Chem. 2024 Mar 21;7:53. doi: 10.1038/s42004-024-01131-4 (PMC10957943; doi:10.1038/s42004-024-01131-4)
Supplement: Supplementary file 1 — Supplementary Information [file 42004_2024_1131_MOESM1_ESM.pdf]

Body odor samples from infants and post-pubertal children differ in their volatile profiles

Diana Owsienko[1], Lisa Goppelt[1], Katharina Hierl[2], Laura Schäfer[2], Ilona Croy[2,3], Helene M. Loos[1, 4*]

[1]Chair of Aroma and Smell Research, Friedrich-Alexander-Universität Erlangen-Nürnberg (FAU), Erlangen, Germany

[2]Department of Psychotherapy and Psychosomatics, Technical University of Dresden, Dresden, Germany

[3]Department of Clinical Psychology, Friedrich-Schiller-University of Jena, Jena, Germany

[4]Fraunhofer Institute for Process Engineering and Packaging IVV, Freising, Germany

*Corresponding Author

E-Mail: Helene.loos@fau.de

Supplementary Note 1

Pilot Study 1 - Selection of target compounds

Aim of the study

Chemical and instrumental analyses are required when it comes to elucidate which body odor (BO) compounds are involved in human chemo-communication and thus may induce a certain behavior in the receiver (e.g., higher pleasantness ratings of infants´ BO compared to post-pubescent BO by parents[1,2]). Different techniques for sampling and isolation of BO compounds have been reported and compared in the field of BO research, whereby headspace techniques (e.g., dynamic headspace, SPME) followed by thermo-desorption, if necessary, are most commonly used. Another possibility is the solvent extraction, which is applicable when textiles are used for sampling. Until now, none of these techniques has been determined as the most suitable one for BO analysis, which is also probably due to the fact that variation of parameters within the methods can lead to differing results. In addition, the choice of the method rather depends on the type of volatiles which are of interest. Gallagher et al. (2008) and Dormont et al. (2013) performed a direct comparison of headspace vs. solvent extraction from BO samples. They pointed out that headspace analysis led to the detection of high-volatile compounds whereas solvent extraction revealed compounds with a higher molecular weight. Further, the detection of carboxylic acids was more challenging when using headspace techniques[3,4]. In this pilot study, we applied different methods to scan a wide range of BO compounds. For this purpose, we first analyzed volatiles in the headspace above worn cotton pads (by adsorption to Twister® with PDMS coating) followed by solvent extraction of the same cotton pads. Moreover, variation in sampling duration was tested (one-night vs. three-night sampling). To prepare for further analyses, results obtained with the two methods were compared to determine target compounds for further quantification.

Supplementary Methods

BO donors and BO sampling

From each age group (AG1 and AG4), three participants were recruited (AG1: M ± SD: 0.3 ± 0.5 years, 100% female; AG4: 16.0 ± 1.6 years, 67% female). The BO samples were collected using 100% cotton T-shirts and bodysuits with pre-treated cotton pads sewed in the axillary area, as described in the main study set up (one-night sampling). These BO samples were used for qualitative analysis/screening.

For quantitative analysis, again, three participants of each age group were recruited (AG1: M ± SD: 1.7 ± 0.5 years, 33% female; AG4: 16.0 ± 0.8 years, 67% female). BO sampling was conducted as described above with a sampling duration of three consecutive nights.

Extraction of volatiles in headspace above samples

Headspace sampling of volatiles from the headspace above the cotton pads occurred via stir bar sorptive extraction (SBSE), using PDMS Twister® (Gerstel GmbH & Co. KG, Mülheim an der Ruhr, Germany). The cotton pads from the left and right axilla were placed inside a glass vial (20 ml) together with a Twister® and closed with a crimp cap. After an extraction time of 75 min at 23°C the Twisters® were analyzed by means of thermal desorption unit (TDU)-GC-MS/O for the occurrence of volatiles and odorants.

Thermal desorption of Twister® and GC-MS/O analysis

The Twister® was thermally desorbed using a Thermal Desorption Unit (TDU) and a MPS 2XL (both from Gerstel GmbH & Co. KG, Mülheim an der Ruhr, Germany). The initial temperature was set at 35 °C and after 30 s of equilibration time the TDU was heated with a rate of 120 °C/min up to 250 °C and held for 5 min. Thermal desorption was performed in splitless mode. The desorbed analytes were transferred with a temperature of 280 °C to the Cooled Injection System (CIS; Gerstel GmbH & Co. KG, Mülheim an der Ruhr, Germany) and cryo-focussed at - 120 °C. After thermodesorption, the CIS was heated up to 240 °C

with a rate of 12 °C/s to transfer the compounds to the GC system, consisting of a Varian 450 GC (Agilent Technologies, Santa Clara, US) equipped with a DB-FFAP column (30 m x 0.25 mm, film thickness of 0.25 µm, J&W Scientific, Agilent Technology, Santa Clara, CA, USA). Helium was used as a carrier gas at a constant flow rate of 2.5 ml/min. The initial oven temperature was kept at 40 °C for 2 min and heated up subsequently to 240 °C with a ramp of 8 °C/min and held for 10 min. Mass spectrometric data was recorded in scan mode (40-500 m/z) with an ionization energy of 70 eV using a Varian Saturn 2200 MS (Agilent Technologies, Santa Clara, US).

Solvent extraction, GC-MS, and GC-O analysis

After TDU-GC-MS analysis solvent extraction was performed with the cotton pads, followed by SAFE and microdistillation as described in the main study set up. Please note that age groups were not pooled, cotton pads of every single participant were analyzed (left and right axilla pooled). The distillates were then analyzed via GC-MS in TIC mode. For further information please see section "Gas chromatography-mass spectrometry (GC-MS)" and "Gas chromatography-olfactometry (GC-O)" in main study/manuscript.

Identification and quantification

During qualitative screening, the compounds were tentatively identified via comparison of MS spectrum and NIST library (match > 800) or using an in-house database established with reference compounds in AMDIS (Version 2.72, National Institute for Standards and Technology, Gaithersburg, USA). In all experiments, blanks (unworn cotton pads) were analyzed in parallel to account for substances originating from the sampling material itself. For the three nights-samples, external calibrations were additionally run.

Statistical analysis

Data were analyzed with IBM SPSS Statistics 25 (IBM Corp. Released 2017. IBM SPSS Statistics for Windows, Version 25.0. Armonk, NY: IBM Corp.) A Mann-Whitney-U test was conducted to compare results obtained for different age groups.

Supplementary Results and Discussion

Qualitative Screening

Headspace analysis revealed 6-methylhept-5-en-2-one (6MHO) and geranyl acetone (GA) among the most abundant volatiles occurring in the BO samples exclusively, see Fig. S1. The average peak areas were compared between the two age groups and a trend for 6MHO was observed, as confirmed with an exact Mann-Whitney-U-test ($U = 0.000$, $p = .100$). The area was about a factor two higher for AG4 (median: 103878) than for AG1 (median: 46893). However, the concentration of all compounds was too low to allow detection by GC-O.

Further, in the distillates, potential difference in the volatile profiles of the two age groups became evident for squalene (SQ), tending to occur in higher quantities in AG4, see Fig. S2.

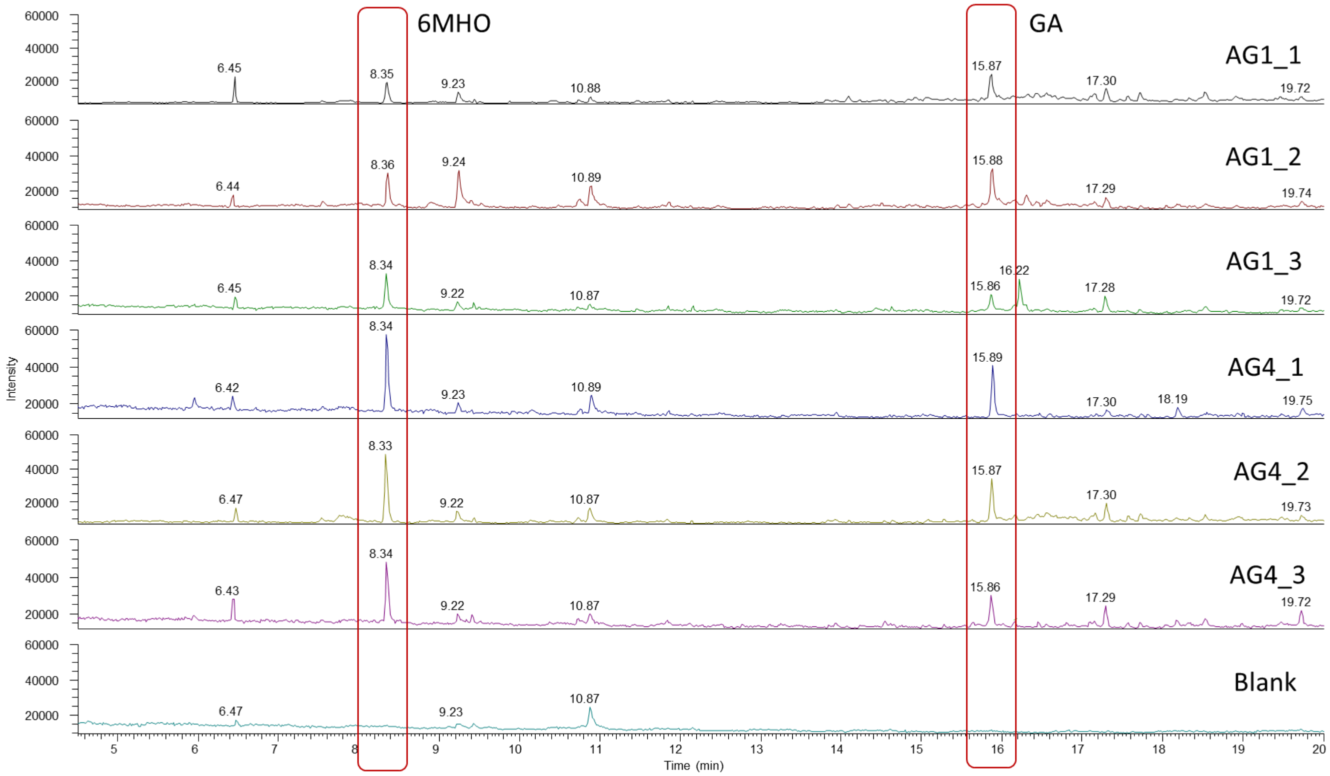

Figure S1. TDU-GC-MS/O measurements after headspace extraction of cotton pads worn during one night by participants of age group 1 (AG1_1, AG1_2, AG1_3) and 4 (AG4_1, AG4_2, AG4_3). A blank (using an unworn pre-treated cotton pad) was analyzed in parallel. Peak at 6.45 min refers to siloxane originating from the PDMS coating of the Twister®. Further peaks: 8.33 min 6MHO, 9.23 min nonanal, 10.88 min decanal, 15.87 GA, 17.30 min 1-dodecanol, 19.75 min 1-tetradecanol. Selected target compounds (6MHO, GA) are marked with red lines.

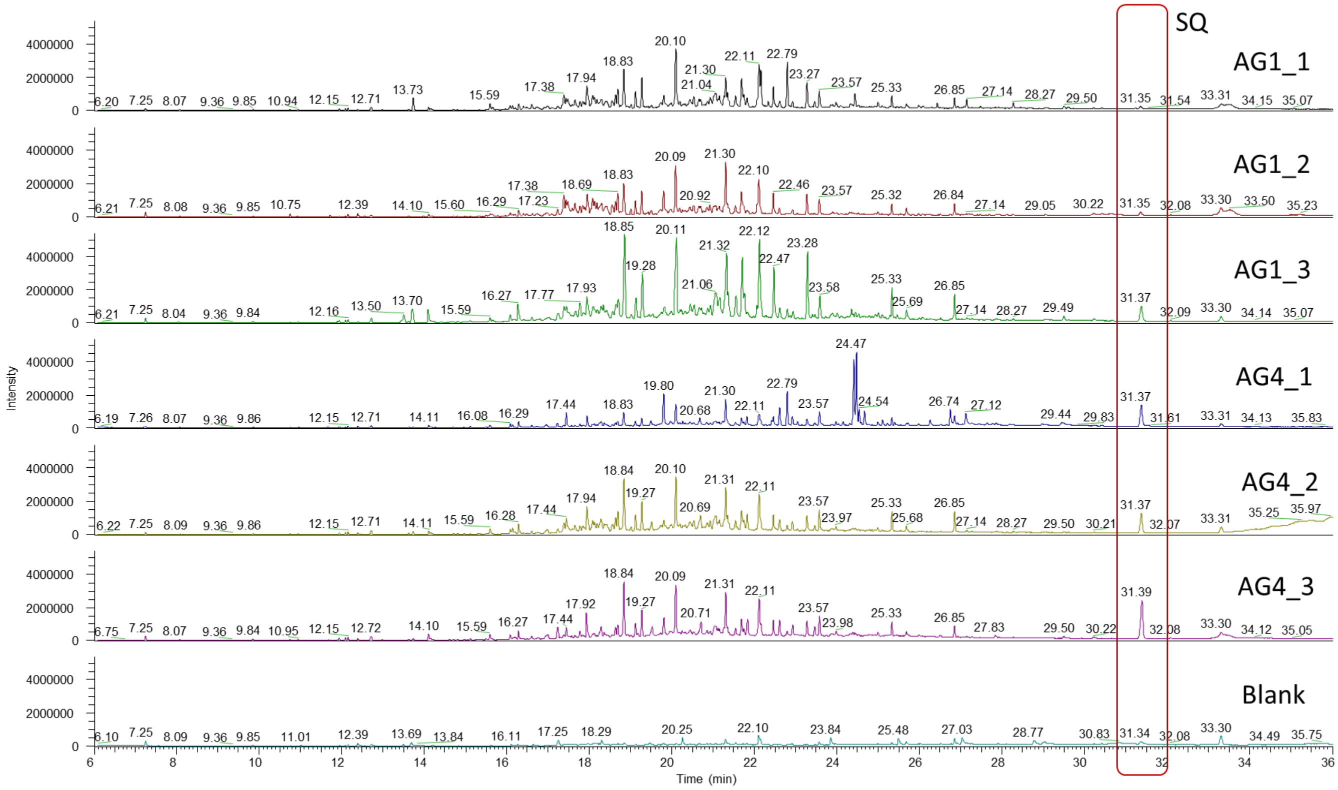

Figure S2. GC-MS measurements after solvent extraction of cotton pads worn during one night by participants of age group 1 (AG1_1, AG1_2, AG1_3) and 4 (AG4_1, AG4_2, AG4_3). A blank (using an unworn pre-treated cotton pad) was analyzed in parallel. Target compound (SQ) is marked with red lines.

Quantitative analysis

For the three-night samples, the following concentration ranges were determined in the distillates by means of external calibration: GA 1.3 – 3.3 µg/ml, SQ 2.6 – 19.5 µg/ml. The ratio of GA to SQ tended to be higher for AG 1 (median: 0.53) compared to AG4 (median: 0.17; exact Mann-Whitney-U-test: U= 0.000, p = .100), see Fig. S3.

Due to a high matrix impact, 6MHO could not be detected in TIC mode, however further experiments showed that detection in SIM mode is possible.

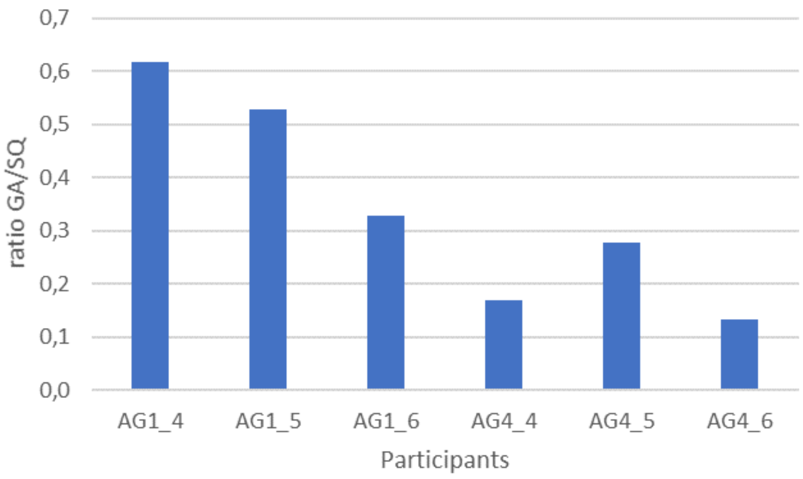

Figure S3. Ratio of concentrations of geranyl acetone (GA) and squalene (SQ).

Conclusion

Headspace sampling above worn cotton pads using Twister® allowed the detection of highly volatile compounds with a low matrix impact, whereas solvent extraction of worn cotton pads revealed a broad range from high to low volatile compounds. Since SQ (low volatile) was only detected by means of solvent extraction and not only absolute concentrations of 6MHO, GA and SQ but also the ratio 6MHO/SQ and GA/SQ were of interest, for the following experiments solvent extraction was the method of choice. Additionally, obtained distillates were suitable for GC-O analysis to investigate odor-active compounds.

Supplementary References

1 Schäfer, L., Sorokowska, A., Sauter, J., Schmidt, A. H. & Croy, I. Body odours as a chemosignal in the mother–child relationship: new insights based on an human leucocyte antigen-genotyped family cohort. *Philos. Trans. R. Soc. B* **375**, 20190266, doi:10.1098/rstb.2019.0266 (2020).

2 Schäfer, L., Sorokowska, A., Weidner, K. & Croy, I. Children's body odors: hints to the development status. *Front. Psychol.* **11**, 320, doi:10.3389/fpsyg.2020.00320 (2020).

3 Gallagher, M. *et al.* Analyses of volatile organic compounds from human skin. *Br. J. Dermatol.* **159**, 780-791, doi:10.1111/j.1365-2133.2008.08748.x (2008).

4 Dormont, L., Bessière, J.-M., McKey, D. & Cohuet, A. New methods for field collection of human skin volatiles and perspectives for their application in the chemical ecology of human–pathogen–vector interactions. *J. Exp. Biol.* **216**, 2783-2788, doi:10.1242/jeb.085936 (2013).

Supplementary Note 2

Table S1. Quantifier ions of target compounds and corresponding internal standards

Target compound	Quantifier	Internal standard	Quantifier
6-methylhept-5-en-2-one	108 m/z	6-methylhept-5-en-2-one d6	113 m/z
geranyl acetone	151 m/z	geraniol d2	123 m/z + 156 m/z
squalene	137 m/z	triacontane	113 m/z

Table S2. Calibration equation and coefficient of determination for the target compounds

Target compounds		Calibration equation	R^2
**6MHO**	Pool 1	y = 1.1343x + 0.0044 (AG1);	0.9996 (AG1);
		y = 1.2044x – 0.0051 (AG4)	0.9992 (AG4)
	Pool 2	y = 1.1161x + 0.0014	0.9998
	Pool 3	y = 1.1073x + 0.0198	0.9997
**GA**	Pool 1	y = 1.0051x + 0.1845 (AG1);	0.9905 (AG1);
		y = 1.2865x + 0.0859 (AG4)	0.9971 (AG4)
	Pool 2	y = 1.2032x + 0.5655	0.9941
	Pool 3	y = 1.2023x + 0.1422	0.9990
**SQ**	Pool 1	y = 2.0728x – 0.5274 (AG1);	0.9955 (AG1);
		y = 2.2994x – 0.673 (AG4)	0.9954 (AG4)
	Pool 2	y = 1.8855x + 0.0864 (AG1);	0.9969 (AG1);
		y = 2.6841x – 0.6738 (AG4)	0.9931 (AG4)
	Pool 3	y = 1.6849x + 0.0436	0.9992

Table S3. Concentrations of target compounds 6-methylhept-5-en-2-one (6MHO), geranyl acetone (GA) and squalene (SQ) in the distillates of pooled body odor samples (BO) as well as room blanks (room) of infants (AG1) and teenagers (AG4).

	**6MHO [µg/ml]**				**GA [µg/ml]**				**SQ [µg/ml]**			
	AG1		AG4		AG1		AG4		AG1		AG4	
	BO	room	BO	room	BO	room	BO	room	BO	room	BO	room
**Pool 1**	2.6	0.4	1.9	0.3	14.9	13.0	38.5	23.5	8040.7	n.d.	53468.9	n.d.
**Pool 2**	2.9	0.8	2.9	0.6	11.5	12.5	21.0	19.1	13094.4	n.d.	104597.2	n.d.
**Pool 3**	2.5	0.5	5.8	0.6	73.3	31.3	50.3	24.7	4167.4	n.d.	71852.0	n.d.

Supplementary Note 3

Pilot Study 2 – Transport of body odor samples

Aim of the study

To gain understanding of the molecular basis of chemical communication in humans, it is essential to conduct research involving on the one hand the isolation and characterization of volatile compounds from body odor (BO) samples and on the other hand the investigation of behavioral responses in the receiver. Only that way, the relationship between BO signatures, transmitted information, and behavioral correlates can be depicted[1]. Such comprehensive research is based on several steps, namely sampling, sensory and behavioral assessment, and chemical analyses of the BOs, and thus requires expertise from different disciplines. In case the collaborating research institutions are geographically distant, a relevant practical question is how to ensure integrity of BO samples during and after transport. Preservation of BO with the help of freezing was investigated by several researchers[2-4]. Results showed in agreement that olfactory properties (e.g., intensity, pleasantness) of the frozen samples were not affected compared to fresh BO samples. Thus, we assumed that different transport conditions of BO samples do not affect their quality, as long as a frozen state of the BO samples can be guaranteed. In the present pilot study, we aimed to experimentally confirm that the quality of BOs is not influenced by different transport conditions. To this aim, we transported the BO samples of 16 children between two laboratories either for few hours with cold packs or overnight by dry ice postal transport and then performed a comparative sensory analysis of these samples by a trained panel.

Supplementary Methods

BO donors

BO donors were 16 children (9 girls and 7 boys) aged between 1.1 years and 17.8 years. The children were divided into four age groups reflecting different stages of development (AG1: 0-3 years, AG2: 4-8 years, AG3: 9-13 years, AG4: 14-18 years; see Table S4) with four children per age group. The participants were recruited during November/December 2019 via postings on the intranet of the University Hospital Carl Gustav Carus, Dresden.

Table S4. Distribution of the odor donor's ages in the different age groups, M = mean value; SD = standard deviation.

**age group**	**1**	**2**	**3**	**4**
**age range in years**	1.1 – 2.8	5.6 – 8.8	9.7 – 13.3	14.9 – 17.8
**M (SD) in years**	1.9 (0.7)	7.1 (1.3)	11.7 (1.3)	16.7 (1.1)

Sampling Procedure

The BO samples were collected using 100% cotton T-shirts and bodysuits (Fruit of the Loom Ltd, Bowling Green, KY, USA). These were washed with fragrance-free detergent (Denkmit Vollwaschmittel Ultra Sensitive, dm-drogerie markt GmbH & Co. KG, Karlsruhe, Germany) and were then worn by the donors for one night. All participants were asked to refrain from using perfumed hygiene products and to avoid strongly spiced food, onions, garlic, leeks, asparagus, cabbage and alcohol for 48 hours before sampling. The donors were provided with an experimental kit containing an odorless washing gel (Eubos Basic Care Liquid Washing Emulsion, Dr. Hobein (Nachf.) GmbH, Meckenheim, Germany) to use before the sampling night and odorless washing powder, same as before, to wash the bed linen. A questionnaire was provided to record the sleeping situation and the hygienic and dietary protocol. After the experimental night, the

BO samples were returned to the experimenter within eight hours and frozen at -20 °C. To prepare the samples for transport, the T-shirts/bodysuits were cut in the middle so that each half contained one armpit. Those halves were then placed in plastic zip bags and frozen for a maximum of 13 days at -20 °C at the University Hospital Carl Gustav Carus, Dresden, Germany.

Transportation

Two different transport routes were applied to the two halves of each body odor sample. During dry ice transport, the samples were transported for about 24 hours in a Styrofoam box filled with dry ice (-78 °C). During transport with thermal packs, the samples were transported in a cool bag together with three cold packs for about 4 hours. The cold packs were previously frozen at -20 °C. The temperature may have risen during the transport, but did not exceed 0 °C, as the cold packs were still solid upon arrival. After the samples arrived at the Chair of Aroma and Smell Research, Friedrich-Alexander-Universität (FAU) Erlangen-Nürnberg, Erlangen, Germany, the sensory evaluation was performed either on the same day or within 24 hours after arrival (samples were stored at -80 °C until evaluation).

Sensory Evaluation

BO raters were 9 trained panelists (5 women, 4 men) of the Chair of Aroma and Smell Research at FAU. Sensory training was performed in weekly sessions and tests by describing odorants with an in-house developed flavor language. To participate in the sensory evaluation an accuracy of > 65% in the tests was required. The panelists were between 25 and 32 years old ($M \pm SD$: 28 ± 3 years).

The samples were kept in the zip bags and thawed 30 min before the evaluation at room temperature. For sensory evaluation, the panelists opened the zip bags, took out the T-shirts/bodysuits and evaluated the odor emanating from the armpit region, to follow the protocol described in [5,6]. The panelists wore gloves during the sensory evaluation.

Sensory profiles were created according to DIN EN ISO 13299:2016. The sensory evaluation was conducted in two sessions. In the first session the total intensity and pleasantness of the samples were rated, and odor attributes were collected by each panelist. Afterwards, the odor attributes were discussed and determined by the panel. The intensities of the selected attributes were then rated in a second session. For each attribute, a reference compound was provided, as follows: sweaty-H (3-hydroxy-3-methylhexanoic acid, aromaLAB GmbH, Martinsried, Germany), sweaty-A (5*S*,8*R*,9*S*,10*S*,13*R*,14*S*)-10,13-dimethyl-1,2,4,5,6,7,8,9,11,12,14,15-dodecahydrocyclopenta[a]phenanthren-3-one (trivial name: 5α-androst-16-en-3-one), Sigma, Steinheim, Germany), soapy (nonanal, Fluka, Steinheim, Germany), fatty ((*E*)-non-2-enal, Aldrich, Steinheim, Germany), flowery ((*E*)-4-(2,6,6-trimethylcyclohexen-1-yl)but-3-en-2-one (trivial name: β-ionone), Aldrich, Steinheim, Germany), vinegar-like (acetic acid, Aldrich, Steinheim, Germany), cotton-like (test fabric, wfk-Testgewebe GmbH, Brüggen, Germany), waxy (decanoic acid, Aldrich, Steinheim, Germany). The samples were rated on a scale of 0 to 10 (0 = not perceptible, 10 = very intense) in terms of total intensity and intensity of specific odor attributes. Additionally, pleasantness was rated on a scale of 0 to 10 (0 = dislike, 5 = neutral, 10 = like). Two samples evinced obvious odorous contaminations (perfume and smoke, age group 1 and 2 respectively). These samples were excluded from further analysis.

Statistical Analysis

The data were analyzed with IBM SPSS Statistics 27 (IBM Corp. Released 2020. IBM SPSS Statistics for Windows, Version 27.0. Armonk, NY: IBM Corp) and JASP 0.14.0.0 (JASP Team (2020). JASP (Version 0.14)[computer software]).

A Bayesian ANOVA was calculated to analyze the probability of the null model (for example there is no effect of transport mode). As the developmental stage affects BO quality[5,7], we furthermore included the respective null models to our analysis (no effect of age group, no interaction of transport mode and age

group). The following effects were modelled: the main effect of transport mode, the main effect of age group, and their interaction effect. We compared each model to the model explaining the data best. Hereby the relative likelihood of the effect can be estimated, without the need to prove the null model.

Within the assessed properties (for instance intensity, pleasantness, sweaty-H, sweaty-A, soapy, fatty, flowery, vinegar-like, cotton-like, waxy), the data were not normally distributed. Due to the robustness of an ANOVA to deliver valid results even with violated assumptions[8,9] and the flexibility of this model for Bayesian testing, we decided to use this method, nonetheless. In total, 10 repeated measures ANOVAs were performed with the independent factors transport mode (2) and age group (4) on the respective odor property serving as target. The main effect of transport mode and the transport mode * age group interaction effect are reported. In addition, the main effect of age was explored. As priors, we assumed an equal likelihood of the null model and the alternative model. The chosen Bayes Factor BF_{10} represents in a ratio of probability how likely it is that the respective model explains the data compared to the model explaining the data best. According to Goss-Sampson et al. (2020) [10], a BF_{10} from 1 to 0.33 can be interpreted as anecdotal, 0.33 to 0.1 as moderate, 0.1 to 0.033 as strong, 0.033 to 0.01 as very strong and under 0.01 as decisive evidence that there is no effect of the respective model.

Supplementary Results

The odor profiles of the BO samples of the two different transport conditions are shown in Fig. S4. Overall, the samples were consistently rated to be of low intensity. The average ratings for the individual attributes ranged between 0.3-0.4 (attributes flowery and vinegar-like) and 1.1-1.3 (cotton-like). The overall intensity of the samples was rated on average 2.8 (transport on dry ice) and 3.0 (transport on cold packs). The odors of the samples were rated to be slightly unpleasant, irrespective of the transport mode. The average ratings are listed in Table S5. The evaluations were consistent across odor donors (see Fig. S5).

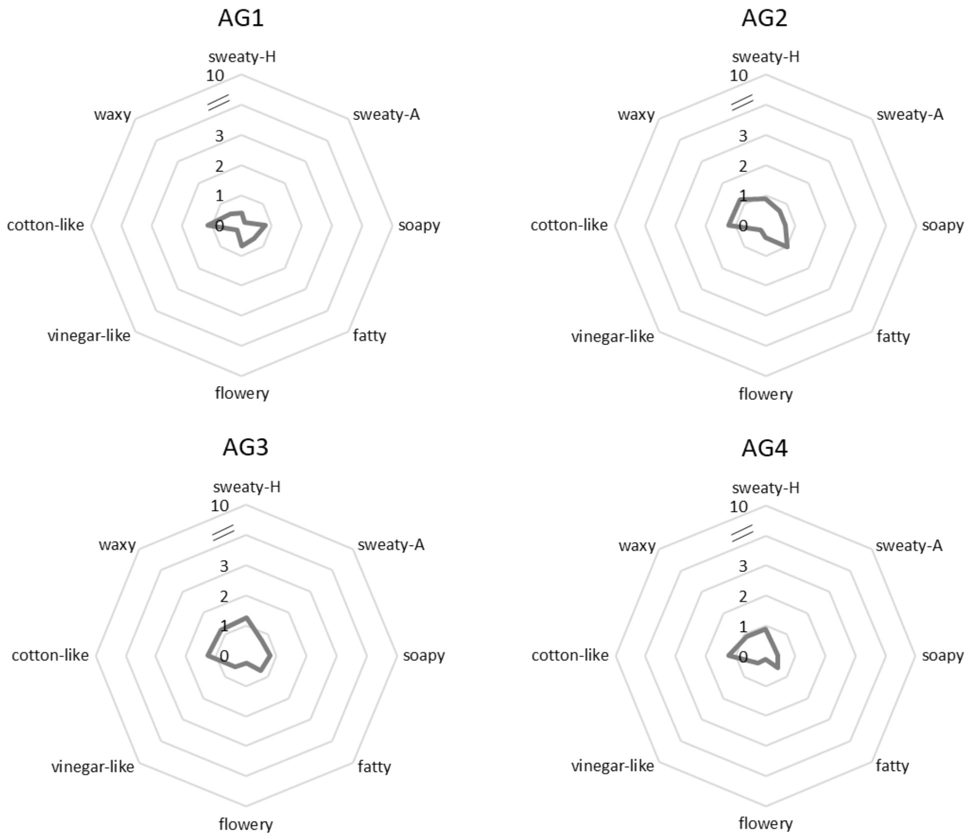

Figure S4. Odor profile of BO samples for each age group. A scale of 0 to 10 (0 = not perceptible, 10 = very intense) was used. AG1: 0-3 years, AG2: 4-8 years, AG3: 9-13 years, AG4: 14-18 years

Table S5. Descriptive statistics of the ratings of the BO samples. Mean values (standard deviations) for each property and both transport modes. The samples were rated by nine panelists on a scale from 0 to 10 (0 = not perceptible, 10 = very intense).

	**intensity**	**sweaty-H**	**soapy**	**flowery**	**cotton-like**
**dry ice**	2.8 (1.6)	1.0 (1.6)	0.8 (1.5)	0.4 (1.0)	1.3 (1.1)
**cold packs**	3.0 (1.6)	0.9 (1.4)	0.4 (0.8)	0.3 (0.9)	1.1 (1.0)
	**pleasantness**	**sweaty-A**	**fatty**	**vinegar-like**	**waxy**
**dry ice**	3.6 (1.7)	0.5 (1.1)	0.7 (1.2)	0.3 (0.8)	0.8 (1.2)
**cold packs**	3.6 (1.6)	0.6 (1.2)	0.7 (1.0)	0.4 (0.9)	1.1 (1.4)

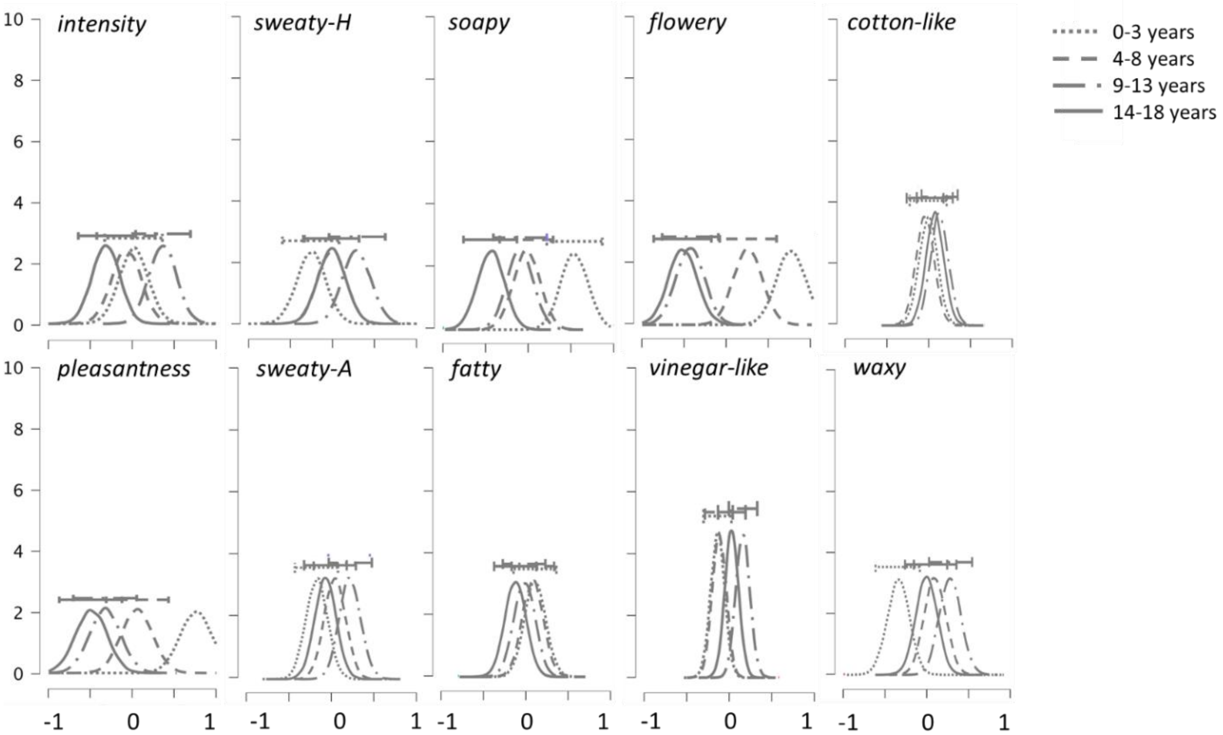

Figure S5. Density functions and scatter plots for the sensory evaluations of BO samples rated for each age group. The samples were rated by nine panelists on a scale from 0 to 10 (0 = not perceptible, 10 = very intense) in terms of total intensity and intensity of specific odor attributes. Additionally, pleasantness was rated on a scale from 0 to 10 (0 = dislike, 5 = neutral, 10 = like). Dot line = 0-3 years, dash line = 4-8 years, dash dot line = 9-13 years, solid line = 14-18 years

The different transport modes did not result in differences in the BO ratings. In line with that, there was no evidence for an effect of transport mode for any of the properties (cf. Table S6). There was moderate evidence against the transport mode model for intensity ($BF_{10} = 0.190$), sweaty-H ($BF_{10} = 0.194$), sweaty-A ($BF_{10} = 0.233$), fatty ($BF_{10} = 0.150$), vinegar-like ($BF_{10} = 0.164$), cotton-like ($BF_{10} = 0.314$) and waxy ($BF_{10} = 0.321$). For the properties pleasantness ($BF_{10} < 0.001$) and soapy ($BF_{10} = 0.006$) and flowery ($BF_{10} < 0.001$), there was decisive evidence against an effect of transportation.

Likewise, the model transport mode * age group did not explain variance in the assessed properties (intensity ($BF_{10} = 0.007$), pleasantness ($BF_{10} = 0.007$), sweaty-H ($BF_{10} = 0.003$), sweaty-A ($BF_{10} = 0.003$),

soapy (BF_{10} = 0.010), fatty (BF_{10} < 0.001), flowery (BF_{10} = 0.016), vinegar-like (BF_{10} = 0.004), cotton-like (BF_{10} < 0.001) and waxy (BF_{10} = 0.031)).

Further, we evaluated our data in terms of the effect of age group. For the properties pleasantness, soapy, flowery and waxy, our analyses suggested that the age group affected the ratings most (see Table S6). Please see Table S7, Fig. S4 and Fig. S5 for details.

Table S6. Probability of the alternative model transport mode and the alternative model transport mode * age group

	**transport mode**		**transport mode * age group**		**best model**
**property**	BF_{10}	interpretation	BF_{10}	interpretation	BF_{10} = 1
**intensity**	0.190	moderate	0.007	decisive	null model
**pleasantness**	6,396e-4	decisive	0.007	decisive	age group
**sweaty-H**	0.149	moderate	0.003	decisive	null model
**sweaty-A**	0.233	moderate	0.003	decisive	null model
**soapy**	0.006	decisive	0.010	very strong	age group
**fatty**	0.150	moderate	3,720e-4	decisive	null model
**flowery**	2,310e-5	decisive	0.016	very strong	age group
**vinegar-like**	0.164	moderate	0.004	decisive	null model
**cotton-like**	0.314	moderate	5,003e-4	decisive	null model
**waxy**	0.321	moderate	0.031	very strong	age group

Table S7. Descriptive statistics of the ratings of the BO samples. Mean values (standard deviations) for each property and each age group. The samples were rated by nine panelists on a scale from 0 to 10. AG1: 0-3 years, AG2: 4-8 years, AG3: 9-13 years, AG4: 14-18 years.

	AG1	AG2	AG3	AG4	AG1	AG2	AG3	AG4
			**intensity**				**pleasantness**	
**dry ice**	3.8 (2.3)	3.4 (2.0)	3.7 (2.0)	2.5 (1.4)	4.7 (1.9)	3.7 (1.7)	3.3 (1.9)	3.2 (1.8)
**cool packs**	4.0 (2.1)	3.4 (1.8)	3.4 (2.0)	2.6 (0.8)	4.4 (2.3)	3.9 (1.7)	3.4 (1.7)	3.2 (1.5)
			**fatty**				**flowery**	
**dry ice**	1.0 (1.7)	0.8 (1.4)	0.8 (1.4)	0.6 (1.0)	1.7 (2.9)	1.1 (2.1)	0.3 (0.8)	0.2 (0.8)
**cool packs**	0.7 (1.1)	0.9 (1.2)	0.6 (1.0)	0.6 (0.8)	1.3 (2.4)	0.8 (1.6)	0.2 (0.5)	0.0 (0.0)
			**sweaty-A**				**soapy**	
**dry ice**	0.1 (0.4)	0.4 (1.0)	0.8 (1.6)	0.4 (0.9)	1.8 (2.4)	0.9 (1.5)	1.1 (1.7)	0.5 (1.1)
**cool packs**	0.5 (1.6)	0.7 (1.6)	0.7 (1.2)	0.4 (0.8)	1.3 (2.0)	0.8 (1.3)	0.5 (1.0)	0.3 (0.5)
			**waxy**				**sweaty-H**	
**dry ice**	0.4 (1.0)	0.8 (1.0)	1.1 (1.5)	0.7 (1.0)	0.4 (1.4)	1.1 (1.5)	1.3 (2.2)	1.1 (1.4)
**cool packs**	0.6 (0.7)	1.1 (1.6)	1.3 (1.6)	1.1 (1.1)	0.9 (1.8)	0.8 (1.2)	1.2 (1.9)	0.7 (1.0)
			**vinegar-like**				**cotton-like**	
**dry ice**	0.2 (0.6)	0.1 (0.6)	0.5 (1.0)	0.3 (0.8)	1.3 (1.2)	1.2 (1.0)	1.3 (1.1)	1.3 (1.0)
**cool packs**	0.2 (0.8)	0.2 (0.7)	0.6 (1.1)	0.4 (1.0)	1.0 (1.2)	1.0 (0.8)	1.3 (1.0)	1.1 (1.0)

Conclusion

In summary, our study confirmed that frozen body odor samples can be transported by the two transport modes applied here without generating different odor profiles. For practical purposes, this implicates that the most convenient of these two transport modes can be chosen depending on individual feasibility.

Supplementary References

1 Smeets, M. A. M. *et al.* Chemical fingerprints of emotional body odor. *Metabolites* **10**, 84, doi:10.3390/metabo10030084 (2020).

2 Roberts, S. C., Gosling, L. M., Carter, V. & Petrie, M. MHC-correlated odour preferences in humans and the use of oral contraceptives. *Proc. R. Soc. B: Biol. Sci.* **275**, 2715-2722, doi:10.1098/rspb.2008.0825 (2008).

3 Lenochova, P., Roberts, S. C. & Havlicek, J. Methods of human body odor sampling: the effect of freezing. *Chem. Senses* **34**, 127-138, doi:10.1093/chemse/bjn067 (2008).

4 Gomes, N., Silva, F. & Semin, G. R. The lasting smell of emotions: the effects of reutilizing fear sweat samples. *Behav. Res. Methods* **52**, 2438-2451, doi:10.3758/s13428-020-01412-5 (2020).

5 Schäfer, L., Sorokowska, A., Sauter, J., Schmidt, A. H. & Croy, I. Body odours as a chemosignal in the mother–child relationship: new insights based on an human leucocyte antigen-genotyped family cohort. *Philos. Trans. R. Soc. B* **375**, 20190266, doi:10.1098/rstb.2019.0266 (2020).

6 Schäfer, L., Sorokowska, A., Weidner, K. & Croy, I. Children's body odors: hints to the development status. *Front. Psychol.* **11**, 320, doi:10.3389/fpsyg.2020.00320 (2020).

7 Croy, I., Frackowiak, T., Hummel, T. & Sorokowska, A. Babies smell wonderful to their parents, teenagers do not: an exploratory questionnaire study on children's age and personal odor ratings in a polish sample. *Chemosens. Percept.* **10**, 81-87, doi:10.1007/s12078-017-9230-x (2017).

8 Glass, G. V., Peckham, P. D. & Sanders, J. R. Consequences of failure to meet assumptions underlying the fixed effects analyses of variance and covariance. *Rev. Educ. Res.* **42**, 237-288, doi:10.3102/00346543042003237 (1972).

9 Lix, L. M., Keselman, J. C. & Keselman, H. J. Consequences of assumption violations revisited: A quantitative review of alternatives to the one-way analysis of variance F test. *Rev. Educ. Res.* **66**, 579-619, doi:10.3102/00346543066004579 (1996).

10 Goss-Sampson, M., van Doorn, J. & Wagenmakers, E. Bayesian inference in JASP: A guide for students. *University of Amsterdam: JASP team* (2020).
